# Clinicopathological and Dermoscopic Baselines in Patients with Lynch Syndrome

**DOI:** 10.3390/cancers15010114

**Published:** 2022-12-24

**Authors:** Giovanni Paolino, Riccardo Pampena, Matteo Riccardo Di Nicola, Santo Raffaele Mercuri

**Affiliations:** Unit of Dermatology and Cosmetology, I.R.C.C.S. San Raffaele Hospital, 20132 Milano, Italy

**Keywords:** Lynch syndrome, dermoscopy, cutaneous lesions, MMR

## Abstract

**Simple Summary:**

Lynch syndrome (LS) is an inherited condition that increases the probability of developing colorectal cancer, endometrial cancer and other malignancies. To date, there are no studies about the dermatological baselines in LS patients; herein, we carried out an observational and monocentric study on the main dermatological features in a population of patients with an established LS, to identify the main clinical and dermoscopic features. This work highlights that the phototype of LS patients reflects the main phototype of the geographic area where the survey was conducted (Italy, with phototype II and III). No specific associations with certain skin manifestations emerged, and the clinical and dermoscopic appearance of the pigmented lesions reflected the features present in the general population and in a control group. There are currently no guidelines for skin screening in LS patients and there is insufficient evidence to ensure increased surveillance in patients with LS.

**Abstract:**

Despite the fact that Lynch Syndrome (LS) patients may also develop extra-colonic malignancies, research evaluating the association between LS and skin cancers is currently very limited. We performed a monocentric clinical and dermoscopic study involving 42 LS patients which referred to the Dermatology Unit for cutaneous screenings. In total, 22 patients showed a mutation in MLH1 and 17 patients a MSH2 mutation. Out of the entire cohort, 83% of LS patients showed brown hairs and 78% brown eyes, and the most frequent phototypes were III and II (respectively, 71.5% and 21%). A positive medical history for an internal malignancy was present in 36% of patients, with colon cancer as the most frequent malignancy in 60% of cases. A total of 853 cutaneous lesions have been analyzed: 47% of patients showed a total number of nevi > 10. The main observed dermoscopic features were a uniform reticular pattern (77% of patients), a mixed pattern (9% of patients) and a uniform dermal pattern (14% of patients). Eruptive cherry angiomas were present in 24% of cases, eruptive seborrheic keratosis in 26% and viral warts in 7% of cases; basal cell carcinoma was detected in 7% of cases. We have not found specific associations with specific skin manifestations, and the clinical and dermoscopic appearance of the pigmented lesions reflected the features present in the general population. To date, there are currently no guidelines for skin screening in LS patients. According to our study, there is insufficient evidence to ensure increased surveillance in LS patients; further studies with larger samples of patients are needed to better investigate dermatological and dermoscopic features in LS carriers.

## 1. Introduction

Lynch syndrome (LS), also called hereditary non-polyposis colorectal cancer, is a rare inherited condition associated with mutations in DNA mismatch repair (MMR) genes [1]. Specifically, LS results from inactivating mutations of DNA mismatch repair (MMR) genes, such as MLH1, MSH2, PMS2 and MSH6 or by a deletion in the EPCAM gene leading to the methylation of the nearby MSH2 promoter [1,2]. These mutations do not allow the system to recognize any DNA mismatches [1,3]. In LS, MLH1 and MSH2 are involved in about 90% of mutation, while MSH6 is involved in 10% of mutations.

Approximately 2–5% of colon–rectal cancers present underlying LS; at the same time, LS patients have the risk of also developing extra-colonic malignancies, if compared to general population, and the cumulative lifetime risks vary according to gender and gene mutation [1]. Several organs (other than colon and rectum) can be involved by the carcinogenic process, such as the endometrium, stomach, kidneys, brain, biliary, pancreas, small bowel, cervix, ovary and the skin. Regarding the skin, the onset of cutaneous neoplasm is mainly related to a variant of LS that is Muir–Torre, characterized by the association between multiple sebaceous tumors (hyperplasia, adenoma and carcinoma) or keratoacanthoma (KA), with one or more visceral carcinomas. However, outside the Muir–Torre variant, in LS currently there are very limited and conflicting data in the literature regarding the association with cutaneous malignancies. Therefore, the association between cutaneous neoplasms in LS patients remains a field to be explored and there is no official protocol on how to perform skin screenings in LS patients.

To date, there are no reports on dermatological baselines in LS patients. We therefore carried out a monocentric investigation on the main dermatological features in patients with LS to identify the main clinical and dermatoscopic characteristics in this class of patients.

## 2. Materials and Methods

From 1 January 2019 to the end of October 2022, a study involving 42 patients with a positive medical history for Lynch Syndrome (LS), categorized according to Amsterdam criteria [4,5], was been performed. Patients were characterized by a family history of HNPCC-associated cancers involving first-degree relatives with at least 2 generations and 1 case diagnosed equal, and less than 49 years [6]. We also included in the current analysis patients with a negative personal history of cancer, but that tested positive for the disease. All patients have been evaluated during the baseline, after 12 months and 36 months of follow-up. The analysis also includes a control group (adjusted by age and gender) with a case-control ratio of 1:1.7; the control group was retrieved from our database concerning patients visited in the same periods as the LS group. Patients negative for LS and/or any other genetic syndromes were included in the control group for the analysis. In order to identify any significant differences between the group and the control, we performed a N-1 Chi-squared test to perform a comparison of proportions. A *p* value < 0.05 was considered statistically significant.

All patients selected for this study were outpatients referred to the Dermatology Unit of the IRCCS San Raffaele Hospital of Milan for routine cutaneous screening. For the evaluation of the cutaneous lesions, the manual dermoscope Heine Delta 20 Tr and the video-dermoscope Vidix 4.0R were used.

Regarding the baseline clinical-pathological features, the following variables have been evaluated: the age of the patients, the gender, the color of their hairs, the phototype (also known as Fitzpatrick’s phototype), oncological familial and personal medical histories, the analysis of cutaneous lesions (both pigmented and non-pigmented) found during the visit and the relative number and dermoscopical aspects of the melanocytic lesions

Regarding melanocytic lesions, during the baseline, each nevus was dermoscopically classified according to a 5-pattern analysis, as reported in 2 previous articles [7,8]: uniform globular, uniform reticular, mixed (central globular or structureless area surrounded by a network or central network or structureless brown-gray area surrounded by a peripheral rim of globules) and finally not defined/unspecified patterns [8,9]. In any case, compared to Gandini et al. [8,10], at baseline, we decided to count the total number of nevi (TN) for each patient, and then categorize them into >10 and ≤10.

## 3. Results

The cohort consisted of 42 patients, 15 male and 27 female. The median age was 47.5 years, ranging between 26 and 81 years (Table 1). The median follow-up of the whole cohort was 36 months, ranging between 14 and 40 months. In total, 25 (*n* = 25) patients showed a mutation in MLH1 and the remaining 17 patients a MSH2 mutation. Out of the entire cohort, 83% of patients (*n* = 35) showed brown hairs and 78% (*n* = 32) brown eyes, with phototype III (*n* = 30; 71.5%) and II (*n* = 9; 21%) as the most frequent phototypes. A positive medical history for an internal malignancy was present in 15 patients (36%), with colon cancer as the most frequent malignancy in 60% of patients, followed by breast (*n* = 2; 13%), prostate cancers (*n* = 1; 6%), ovary (*n* = 1; 6%), gastric cancer (*n* = 1; 6%) and cervix (*n* = 1; 6%). The remaining 64% of patients did not have a positive clinical history for cancer, but they performed periodic follow-up checks for preventive purposes only.

**Figure 1:**
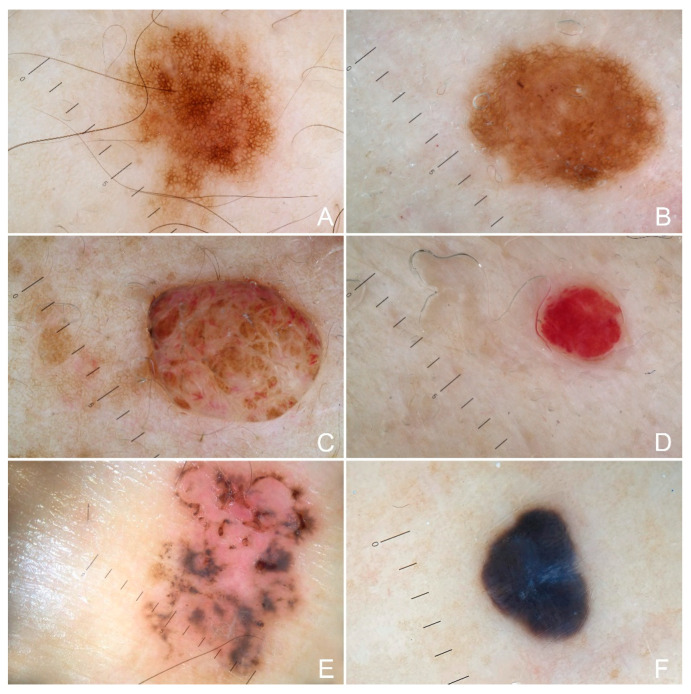
Cutaneous lesions observed in patients with Lynch syndrome (LS) (**A**)**.** A pigmented lesion with a typical uniform reticular pattern; (**B**) a pigmented lesion with a mixed pattern composed of a central structureless area surrounded by a network; (**C**) a dermal nevus showing a uniform and unspecified dermal pattern; (**D**) a cherry angioma with uniform reddish pattern; (**E**), pigmented basal cell carcinoma and (**F**) uniform dermal pattern in a blue nevus.

**Figure 2:**
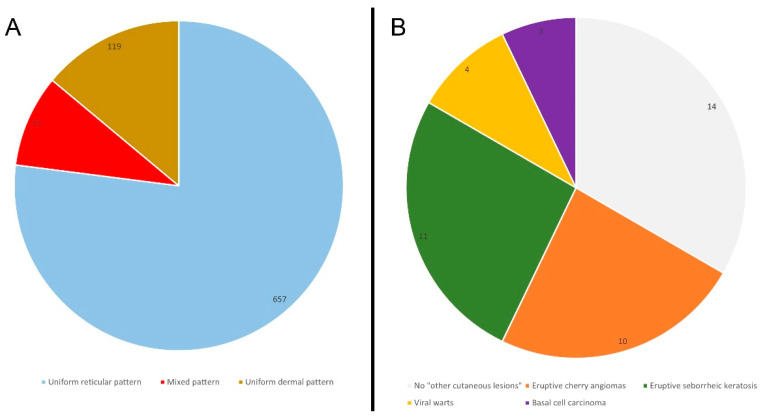
Main dermoscopic features observed in the 853 analyzed cutaneous pigmented lesions (**A**) and other clinical cutaneous lesions observed in the sample (**B**).

A total of 853 cutaneous pigmented lesions have been analyzed in LS patients; specifically, 45% of patients (*n* = 19) showed a total number of nevi > 10, while regarding the main observed dermoscopic features, a uniform reticular pattern was observed in 77% of the analyzed pigmented lesions (*n* = 657), followed by a mixed pattern (central globular or structureless area surrounded by a network) in 9% of cases (*n* = 77) and uniform dermal pattern in 14% of cases (*n* = 119). (Figure 1) Other cutaneous lesions were present in 67% of the patients (*n* = 28); in particular, eruptive (>10) cherry angiomas were present in 24% of the patients (*n* = 10), eruptive seborrheic keratosis in 26% (*n* = 11) and viral warts in 10% (*n* = 4). During a follow-up of 36 months, a basal cell carcinoma was detected in 7% of cases (*n* = 3), specifically, after 13, 23 and 36 months of follow-up (Figure 2) No melanocytic lesions have been excised. No significant differences have been identified between the clinicopathological and dermatoscopic baselines between LS patients with MLH1 and MSH2 mutations. Finally, the main clinical-pathological features of a control group (adjusted by age and gender) have been reported (Table 1). The control was characterized by 70 patients (42 female and 28 male), with a median age of 48 years (ranging between 22 years and 85 years). No significant differences emerged between LS patients and the control group based on the clinical-pathological variables analyzed, except for the general incidence of a positive personal cancer history that, as expected, was higher in the LS group (36%) than in the control (7%) (Table 1; *p* < 0.001).

## 4. Discussion

The spectrum of extra-colonic malignancies in LS patients involves several organs (such as stomach, kidney, brain, biliary tract, pancreas and small bowel), including the skin. In this regard, the skin, being the largest human organ, can be subject to various mutations, which can cause tumor lesions [8]. Therefore, understanding the cutaneous clinicopathological baselines in this class of patients is important for both dermatologists and other specialists involved in the study and management of LS.

Considering that the general incidence of LS is 1: 660–2000 people, and that in Italy LS is present in 2–3% of colon–rectal cancers, the analysis of our case study is justified. In this regard, therefore, identifying additional risk factors for the eventual development of extra-colonic cancers is important for the carriers. First of all, in our sample, 83% of patients (*n* = 35) showed brown hairs and 78% (*n* = 32) brown eyes, with phototype III as the most represented phototype (71.5%), followed by phototype II (21%). At the same time, phototype III was also the most represented in the control group (70%), as well as brown hairs (82%) and brown eyes (74%) (Table 1). As observed also for BRCA patients [8], the prevalence of phototype III in LS patients can be explained by the greater prevalence of people with brown hair and eyes in Italy. Accordingly, the phototype of LS mutation carriers is conditioned by the most widespread phototype in the place where the study was carried out (i.e., Italy). In particular, the phototype of LS carriers is normal, unlike MCR1R or CDKN2A patients, who tend to have red hair or multiple dysplastic nevi, respectively [8].

Concerning melanocytic lesions, in both the group and control (Table 1), the age-related prevalence of dermoscopic patterns in acquired melanocytic nevi (reticular pattern being the main prevalent pattern) was in line with those of the general population [8,9,10]. Regarding the incidence of cutaneous malignancies, in a follow-up of 36 months, we detected 3 basal cell carcinomas (BCC) in LS patients (including 1 rare case in which BCC arose and localized in the foot) and 5 BCC in the control group. We did not detect other cutaneous malignancies in LS patients, and, contrariwise to BRCA carriers, we did not detect a high incidence of eruptive cherry angiomas (eCAs). According to the literature [11,12,13,14,15,16,17], sebaceous adenoma, sebaceous epithelioma, sebaceous adenocarcinoma, keratoacanthoma and squamous cell carcinoma are the cutaneous cancers mostly associated with the variant of LS called Muir–Torre. The absence of these neoplasms in our sample is justified by the absence of Muir–Torre syndrome in our cohort. Indeed, contrariwise to Muir–Torre syndrome, the association between pure LS and skin cancers is more sporadic and not well defined, with conflicting results in the literature. BCC, cutaneous squamous cell carcinomas and cutaneous sarcoma have been described in a family with LS [18], but due to the small sample number the impact of this result is limited. In a study involving 121 LS families, among extracolonic cancers (*n* = 282), the authors detected 1 sebaceous adenocarcinoma and 11 other skin cancers that the authors did not specify in the manuscript [19]. At the same time, in a study evaluating the frequency of extra-colonic tumors in 60 unrelated Brazilian LS families, among 204 extracolonic cancers, a cutaneous neoplasm was present in 8 cases [1]. Accordingly, in LS the incidence of cutaneous neoplasms is about 3% to 5%, among extracolonic cancers. The detection of 3 BCC in our sample (and 5 cases in control group), that corresponds to 7% of cases, seems to confirm the literature. To date, according to the literature [1,14,18] and to our study, the incidence of cutaneous neoplasms in LS patients is similar to the incidence in general population in Italy [19]; therefore, it remains to be confirmed whether there is a true increased risk of skin cancer in patients with LS, outside of the Muir–Torre variant. 

A strength of this study is the definition of the general cutaneous clinical-pathological baselines and dermoscopic features in a sample of LS patients, in a monocentric study. A limitation of the current study was the small sample size; however, LS is a rare genetic mutation, and it might be hard to collect larger samples of LS mutation carriers in a single center.

## 5. Conclusions

This work highlights that the phototype of LS patients reflects the main phototype of the geographic area where the survey was conducted (i.e., phototype II and III, which are the main phototypes in Italy). Compared to other mutations (e.g., BRCA carriers), we have not found specific associations with certain skin manifestations (such as eCAs), and the clinical and dermoscopic appearance of the pigmented lesions reflect the features present in the general population (as also confirmed by the control group). There are currently no guidelines for skin screening in LS patients. Furthermore, there is insufficient evidence to ensure increased surveillance in patients with LS or with a positive family history for this condition. Further studies with larger samples of patients are needed to better investigate dermatological and dermoscopic features in LS carriers.

## Figures and Tables

**Table 1:** Clinicopathologic features of the sample.

	*n*	%	cn	c%	*p* *
Gender					NS
Male	15	35.7	28	40	
Female	27	64.3	42	60	
Hairs					NS
Blond	3	7.1	6	8.5	
Brown	35	83.3	57	82	
Black	4	9.5	7	10	
Eyes					NS
Blue	6	14.3	9	13	
Brown	31	73.8	52	74	
Black	3	7.1	5	7	
Green	2	4.8	4	6	
FP					NS
I	1	2.4	3	4	
II	9	21.4	15	21	
III	30	71.4	49	70	
IV	2	4.8	3	4	
PCH					*<0.001*
Positive *	15	36	5	7	
Negative	27	64	65	93	
CL					NS
None	4	9.5	12	17	
Positive	38	90.5	58	83	
TN					NS
>10	19	47	34	49	
≤10	23	53	36	51	
Nevi pattern					NS
R	657	77	1.118	74	
MC	25	2.9	61	4	
MP	32	3.7	45	3	
G	20	2.3	61	4	
U	119	14	227	15	

FP means Fitzpatrick’s phototype; * positive personal cancer history (PCH)*: colon cancer 9, breast 2, prostate cancer 1, ovary 1, gastric cancer 1 and cervix *n* = 1; cutaneous lesions observed (CL), including more lesions in the same patients: eruptive cherry angiomas (eCAs) 10, seborrheic keratosis 11, basal cell carcinoma 3, psoriasis 2, sebaceous cysts 3, warts 4, condyloma 1, accessory nipple 1, actinic keratosis 2. Positive personal cancer history (PCH) in healthy (cn) control group: breast 3, prostate cancer 2; cutaneous lesions observed (CL), including more lesions in the same patients in the healthy control group: eruptive cherry angiomas (eCAs) 28, seborrheic keratosis 35, basal cell carcinoma 5, psoriasis 3, sebaceous cysts 1, actinic keratosis 8. TN means total number of nevi, with a mean number of nevi of 20 (ranging between 5 and 25); Nevi pattern: uniform globular pattern (G), reticular pattern (R), mixed pattern composed of central globular or structureless area surrounded by a network (MC), mixed pattern composed of a central network or structureless brown-gray area surrounded by a peripheral rim of small brown globules (MP) and unspecified pattern (U). ≠ The analysis of the nevi pattern in the group was performed on a total of 853 melanocytic lesions that have been evaluated in all 42 patients, while the analysis in the healthy control group was performed on 1.512 melanocytic lesions that have been evaluated in all 70 patients. p* N-1 Chi-squared test to perform a comparison of proportions; in *Italic* significant values; NS means not statistically significant.

## Data Availability

Not applicable.
